# Electro-acupuncture for post-stroke cognitive impairment: A protocol for systematic review and meta-analysis

**DOI:** 10.1097/MD.0000000000028774

**Published:** 2022-02-25

**Authors:** Guanli Xie, Xiaoxia Tang, Wen Luo, Yanfei Xu, Deguang Li, Zhe Wang, Changfei Yuan, Yibo Xia, Xiaohan Zhou, Miao Tian, Zhifa Yuan, Tao Wang, Jianglong Liao

**Affiliations:** a Yunnan University of Chinese Medicine, Kunming, Yunnan, China; b Kunming Municipal Hospital of Traditional Chinese Medicine, Kunming, Yunnan, China; c Kunming Municipal Hospital of Traditional Chinese Medicine & Kunming Combination of Chinese and Western Medicine Minimally Invasive Spine Technology Center, Kunming, Yunnan, China.

**Keywords:** cognitive impairment, electro-acupuncture, protocol, post-stroke cognitive impairment, review, stroke

## Abstract

**Background:**

A considerable number of stroke survivors suffered from cognitive impairment, and more than one third of stroke survivors are affected at 3 and 12 months after the stroke. Although the published systematic reviews suggest that acupuncture can help improve post-stroke cognitive dysfunction, the power of the results is low due to study limitations. Therefore, this review is necessary to analyze the effect of acupuncture on cognitive impairment after stroke and to provide evidence for cognitive impairment in stroke.

**Methods:**

This study will be carried out in strict accordance with the Cochrane Handbook for Systematic Reviews of Interventions. According to the pre-established search strategy (PICOS framework), all the literature will be obtained from online databases including Cochrane Central Register of Controlled Trials in the Cochrane Library, Medline (via PubMed), EMBASE (via embase.com), CINAHL (via EBSCOhost), China National Knowledge Infrastructure database, WanFang Database, Chinese Science and Technology Periodical Database, and Sino-Med Database from inception until December 31, 2021 with no language limitations. Two reviewers will screen the records and include quality studies according to inclusion criteria independently. The data needed will be extracted independently by 2 authors according to a table of data extraction. Any inconsistencies in literature screening and data collection will be resolved to reach a consensus via discussion with a third author. Risk of bias for each study will be assessed using risk of bias tool. RevMan5.3 will be used to analyze the data. Heterogeneity will be identified and measured by Chi^2^. Subgroup analyses and sensitivity analysis will be carried out. Grading of Recommendations Assessment, Development and Evaluation will be used to evaluate the evidence for each outcome.

**Conclusion:**

The results of this study will provide a theoretical basis for the clinical use of electro-acupuncture to treat cognitive dysfunction after stroke.

**Unique INPLASY number:**

INPLASY202210038

## 1 Introduction

### 1.1 Description of condition

A considerable number of stroke survivors suffered from cognitive impairment, and more than one third of stroke survivors are affected at 3 and 12 months after the stroke.^[1,2]^ Although the reported results show that the prevalence of cognitive dysfunction after stroke is not the same in different regions of the world, the data still show that cognitive dysfunction after stroke is a very common problem. The prevalence of the cognitive impairment 3 months after stroke ranges from 21.8% to 69.8%,^[3–10]^ and 44% to 70% after 6 months.^[11,12]^ Even years after the stroke, cognitive dysfunction still plagues stroke patients. It was reported that 34% to 57% stroke patients suffered from the cognitive impairment during the first year after stroke.^[12,13]^ What is more, study showed that poststroke cognitive impairment (PSCI) is common even after successful clinical recovery.^[14]^ Cognitive dysfunction affects stroke patients for many years^[1,2]^ and are associated with poor long term survival, rates of disability.^[15]^ The study found that the RR associated with dependent life and cognitive impairment was 2.4 after adjusting for physical injury and age 3 months after stroke.^[16]^ According to the Guidelines for Adult Stroke Rehabilitation and Recovery, screening for cognitive deficits is recommended for all stroke patients before discharge home (I, Level of Evidence B) and non-drug therapies for cognitive impairment, including memory is recommended.^[15]^

### 1.2 Description of intervention

#### 1.2.1 How does acupuncture affect PSCI

As one of the most important treatment methods of Traditional Chinese Medicine, acupuncture has a history of more than 2000 years and plays an important role in protecting, maintaining and restoring health.^[17]^ In clinical practice, acupuncture is also widely used to improve cognitive dysfunction after stroke. Over the past few decades, there are many clinical studies have evaluated the clinical validity and safety of acupuncture in patients with cognitive dysfunction, especially in patients with cerebrovascular dementia and cognitive dysfunction after stroke. And the result of systematic reviews of randomized controlled trials (RCTs) showed that acupuncture had positive effects on cognitive function after stroke^[18,19]^.

#### 1.2.2 The importance of this review

At present, PSCI has become a hot spot in international stroke research and intervention. With the aggravation of population aging, the incidence of PSCI is increasing, which brings heavy economic burden to the society and family. How to better treat for PSCI is an important issue in the medical community. Acupuncture was used widely for patients with PSCI. Although the published systematic reviews suggest that acupuncture can help improve post-stroke cognitive dysfunction, the power of the results is low due to study limitations.^[18,19]^ The review reported by Liu et al. included 21 trails, but only 9 studies reported the primary outcome (cognitive function), and the cognitive function was measured using different studies.^[18]^ And the largest sample size in the study reported by Liu is only 40 cases, which also makes the power of the study results low.^[18]^ The review reported by Zhou included 37 studies with 2869 patients. However, studies in which acupuncture was used in combination with other interventions as an intervention package in the experimental group were also included, which makes the power is low. Therefore, this review is necessary to analyze the effect of electro-acupuncture for PSCI and to provide evidence for the using of electro-acupuncture therapy for PSCI.

## 2 Objectives

The aim of this study is to analyze the effect of acupuncture on cognitive impairment and to provide evidence for using acupuncture for PSCI.

## 3 Methods

This protocol is reported according to the Preferred Reporting Items for Systematic Review and Meta-Analysis Protocols 2015 Checklist.^[20]^ The details of the PRISMA-P of this study can be obtained online supplementary Digital Content S1. This protocol has been registered on the INPLASY website (registration number: INPLASY202210038: https://inplasy.com/inplasy-2022-1-0038/).

### 3.1 Selection criteria

#### 3.1.1 Type of subjects

All stroke patients will be included regardless of the type of stroke (hemorrhagic or ischemic stroke), age, gender, severity, course of the disease (acute stage, subacute stage or chronic stage) and region. The diagnose of stroke must be according to the World Health Organization (WHO) definition,^[21]^ or a clinical definition of stroke when the WHO definition was not specifically stated, or confirmed by MRI or CT. Any study including participants with other type of injury (such as multiple sclerosis traumatic brain injury or spinal cord injury) will be excluded.

#### 3.1.2 Type of intervention

All studies using electro-acupuncture as an intervention of the experimental group for stroke will be included in the study. Acupuncture points, intensity, time, intervention cycle are not limited. Studies that using electro-acupuncture combined with Traditional Chinese medicine or western medicine as intervention for the experimental group will be excluded. Studies using scalp acupuncture or manual acupuncture as intervention for the experimental group will be excluded. The intervention methods used in the control group were not restricted. Studies that only compared different methods of electro-acupuncture (such as different points, different frequencies or different intensities, etc.) or only compared electro-acupuncture with traditional Chinese medicines will be excluded in this study.

#### 3.1.3 Type of outcomes

##### 3.1.3.1 Primary outcomes

Cognitive function will be defined as primary outcome regardless of the instruments including Mini-Mental State Examination (MMSE), Montreal Cognitive Assessment (MoCA), Neurobehavioral Cognitive Status Examination, (NCSE), Loewenstein (LOTCA), auditory verbal learning test (AVLT), Stroop color and word test (SCWT), trail making test (TMT), Boston naming test (BNT), digital span test (DST), clinical dementia rating scale (CDR), Wechsler memory scales (WMS), etc.

##### 3.1.3.2 Secondary outcomes

The secondary outcome measures of this review will include activities of daily living reported in the studies. Possible measure instruments for consideration include global assessment tools of ADL such as: Modified Barthel Index (MBI), Barthel Index (BI), Rivermead ADL Assessment, Modified Rankin Scale, Functional Independence Measure (FIM), etc. Other secondary outcomes include drop out from the study during the treatment phase, and adverse events (including death from all causes).

#### 3.1.4 Type of studies

All randomized controlled trials (RCTs) focused on this topic will be included regardless of publication status or publication type. Other type of studies such as non-RCTs (including letter to editors, review, case report, ect.), quasi-RCTs will not be included, For the randomized controlled cross-over trials, only the first period will be considered as a parallel group trial. The language of publication will be limited to English and Chinese without any restrictions on publication type.

### 3.2 Search methods

All the literature will be obtained from online databases via systematic research from inception until December 31, 2021 with no language limitations. Online databases include Cochrane Central Register of Controlled Trials in the Cochrane Library, Medline (via PubMed), EMBASE (via embase.com), CINAHL (via EBSCOhost), China National Knowledge Infrastructure database, WanFang Database, Chinese Science and Technology Periodical Database, and Sino-Med Database. The English terms were used individually or combined “cerebrovascular disorders,” “cognition disorders,” “memory disorders,” “acupuncture,” “electroacupuncture,”and the Chinese searching terms were “nao cu zhong (stroke),” “nao geng si (cerebral infarction),” “ren zhi gong neng (cognitive function),” “zhen ci (acupuncture),” “dian zhen (electroacupuncture),”. The search strategies we have built for Medline via PubMed as showed in Table 1. The retrieval strategy of other databases will be adjusted and modified based on this.

**Table 1:** Search strategy for Medline via PubMed.

Number	Search items
#1	“cerebrovascular disorders” [Mesh] or “brain injuries’ ’[Mesh] or “brain injury, chronic” [Mesh]
#2	(“stroke$” or “cva” or “poststroke” or “post-stroke”) [tw]
#3	(“cerebrovasc$” or “cerebral vascular”) [tw]
#4	(“cerebral” or “cerebellar” or “brain$” or “vertebrobasilar”) [tw]
#5	(“infarct$” or “isch?emi$” or “thrombo$” or “emboli$” or “apoplexy”) [tw]
#6	#4 and #5
#7	(“cerebral” or “brain” or “subarachnoid”) [tw]
#8	(“haemorrhage” or “hemorrhage” or “haematoma” or “hematoma” or “bleed$”) [tw]
#9	#7 and #8
#10	“hemiplegia” [Mesh] or “paresis” [Mesh]
#11	(“hempar$” or “hemipleg$” or “brain injur$”) [tw]
#12	“Gait Disorders, Neurologic”[Mesh]
#13	#1 or #2 or #3 or #6 or #9 or #10 or #11 or #12
#14	“cognition disorders” [Mesh] or “confusion” [Mesh] or “neurobehavior almanifestations” [Mesh] or “memory disorders” [Mesh]
#15	(“agnosia” or “amnesia” or “confusion” or “inattention”)[tw]
#16	“cognition” [Mesh] or “Arousal” [Mesh] or “Orientation”[Mesh] or “Attention”[Mesh] or “memory”[Mesh] or “perception”[Mesh] or “mental processes” [Mesh] or “thinking” [Mesh] or “Concept Formation”[Mesh] or “Algorithms” [Mesh] or “Recognition (Psychology)” [Mesh] or “Judgment” [Mesh] or “Awareness” [Mesh] or “Problem Solving” [Mesh] or “Generalization (Psychology)” [Mesh] or “Transfer (Psychology)” [Mesh] or “comprehension”[Mesh] or “Impulsive Behavior” [Mesh] or “Learning”[Mesh]
#17	((“cogniti$” or “arous$” or “orientat$” or “attention$” or “concentrat$” or “memor$” or “recall” or “percept$” or “think$” or “sequenc$” or “algorithm$” or “judg? ment$” or “awareness” or “problem solving” or “generali?ation” or “transfer” or “comprehension” or “learning”) adj10 (“disorder$” or “declin$” or “dysfunct$” or “impair$” or “deficit$” or “abilit$” or “problem$”)) [tw]
#18	(“dysexecutive syndrome$” or “mental process$” or (“concept” adj5 “formation”) or “impulsive behavio? r$” or “executive function$”) [tw]
#19	#14 or #15 or #16 or #17 or #18
#20	#13 and #19
#21	“acupuncture” [Mesh]
#22	“acupuncture” or “electroacupuncture” or “electro-acupuncture” [tw]
#23	#21 or #22
#24	randomized controlled trial [pt]
#25	controlled clinical trial [pt]
#26	randomized [tiab]
#27	placebo [tiab]
#28	clinical trials as topic [mesh: noexp]
#29	randomly [tiab]
#30	trial [ti]
#31	#24 OR #25 OR #26 OR #27 OR #28 OR #29 OR #30
#32	human [mh]
#25	#20 and #23 and #31 and #32

### 3.3 Additional resources

Additional data can be obtained from references that have been included in the study.

### 3.4 Data collection and analysis

#### 3.4.1 Literature screening

**Figure 1:**
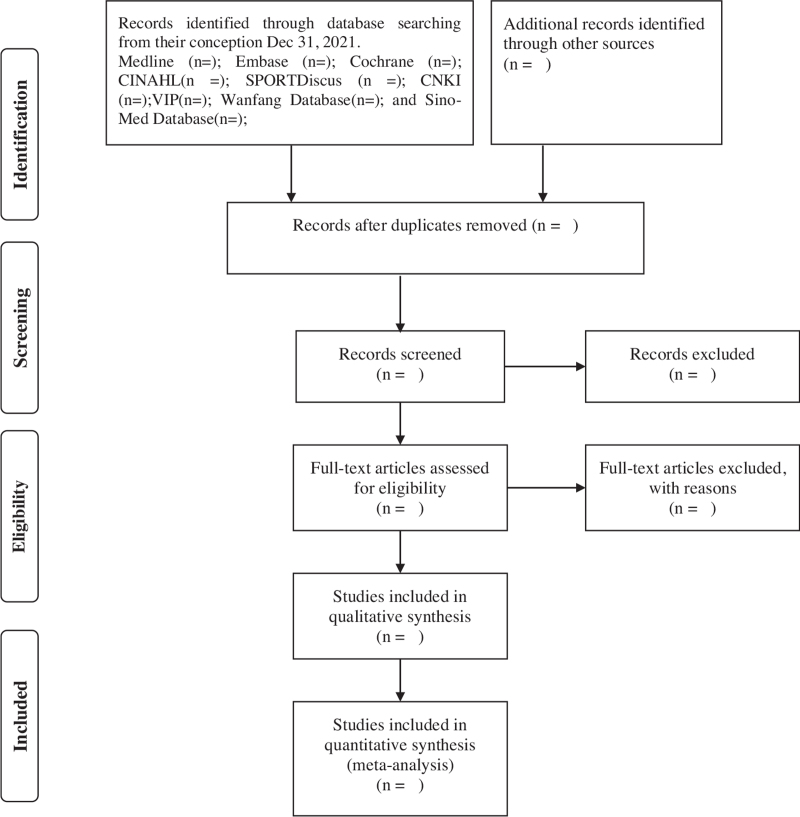
Flow diagram of the study selection process.

The literature management software, EndNote X7, will be used to manage the records collected. The PRISMA flow diagram (Fig. 1) will be used to guide screen literature report the details in the results of the study. In short, literature screening is divided into the following steps:

1. Delete duplicate records.
2. Identify potentially eligible literature after screen titles and abstracts of records.
3. Include eligible studies after reading full text by 2 reviewers independently based on inclusion criteria (the subject, intervention, study design strategy).

If necessary, the original author will be contacted to obtain sufficient information to help us make a judgment. Any disagreements in judgement will be resolved to reach a consensus via discussion with a third author.

#### 3.4.2 Data extraction and management

The data needed for the study will be extracted independently by 2 authors according to a table of data extraction. The extracted data includes the following parts:

1. Basic information including the author, title, journal, publish year, etc.;
2. Information about the subject including species, age, weight, feeding conditions, animal models, criteria used for including and excluding animals, etc.;
3. Intervention information including intervention for experimental group and the intervention for control group, intervention points, intensity of intervention, frequency of intervention, treatment cycle, etc.;
4. outcomes information such as the instruments used in studies.
5. study design: for example, experimental group settings, number of experimental groups, number of animals per groups.

For continuous outcomes such as mean and standard deviation or standard error will be extracted. For counting data such as fatality, the number of events will be extracted. For those studies with multiple experimental groups or multiple control groups, the data will be divided into several pairs (experimental groups vs. control group). Microsoft Excel will be used to extract and manage data. RevMan5.3 will be used to analyze the data. Any inconsistencies in data collection will be resolved to reach a consensus via discussion with a third author

#### 3.4.3 Assessment of risk of bias

Quality assessment of included studies will be carried out independently by 2 authors independently. The Cochrane Risk of Bias tool–version 2 (RoB 2)^[22]^ will be used to assess the methodological quality of the included studies. Following domains will be assessed: randomization process, intended interventions, missing outcome data, measurement of outcomes, and selective reporting of results. Each included study will be assessed as having a high risk of bias, unclear risk of bias or low risk of bias for each entry. Any inconsistencies will be resolved to reach a consensus via discussion with a third author.

#### 3.4.4 Missing data processing

If there are difficulties in making a judgment in any of the above steps due to unclear or insufficient data, authors of the original studies will be contacted to get sufficient data. If sufficient data is not available after multiple efforts, the impact of missing data on the results will be discussed in the discussion section.

#### 3.4.5 Identifying and measuring heterogeneity

According to Cochrane Handbook for Systematic Reviews of Interventions, clinical diversity (clinical heterogeneity), methodological diversity (methodological heterogeneity), and statistical heterogeneity should be considered in studies.^[23]^ Heterogeneity will be identified and measured by Chi-Squared test (*P* < and *I*^2^ statistic. A rough guide to interpretation in the context of meta-analyses is as followings:

1. 0% to 40%: might not be important;
2. 30% to 60%: may represent moderate heterogeneity;
3. 50% to 90%: may represent substantial heterogeneity;
4. 75% to 100%: considerable heterogeneity.

If the results suggest that there are significant heterogeneity between included studies, appropriate measures will be taken to addressed heterogeneity including but not limited to check again the data are correct, do not do a meta-analysis, explore heterogeneity, perform a random-effects meta-analysis, meta-regression and subgroup analysis.

#### 3.4.6 Detection of publication bias

If more than 10 of the included studies reported the same outcome, publication bias will be carried out for each outcome.^[23]^ A scatter plot of the estimate of effect from each study in the meta-analysis, funnel plots, will be used to detect the publishing bias. The final results will be discussed in detail in the discussion section.

#### 3.4.7 Analysing data

Meta-analysis will be performed to describe the effect of electro-acupuncture for PSCI. The parameters will be set based on the outcome type, heterogeneity results. Mean difference (SD) or the standardized mean difference (SMD) will be used for meta-analysis of continuous data. Odds ratios, risk ratios or risk differences will be used for meta-analysis of dichotomous data. Random-effect model or fixed-effect model will be used base on heterogeneity results. Meta-analyses will be illustrated using a forest plot.

#### 3.4.8 Subgroup analyses

Subgroup analyses will be done as a mean of investigating heterogeneous results or to answer specific questions about types of acupuncture or different acu-points used in studies. Subgroup analyses will be done for subsets of subjects, subsets of acupuncture points (head acupuncture points, limbs acupuncture points, body acupuncture point).

#### 3.4.9 Sensitivity analysis

In order to ask the question, ‘Are the findings robust to the decisions made in the process of obtain them’, a sensitivity analysis will be performed. It may involve undertaking meta-analysis twice:

1. including all the studies;
2. including only these that are definitely known to be eligible.

The influence of articles not included in the second analysis on the results will be discussed in the results report of this study.

#### 3.4.10 Grading the quality of evidence

The quality of evidence for each outcome will be analyzed using Grading of Recommendations Assessment, Development and Evaluation system.^[24]^

## 4 Discussion

PSCI is a common complication after stroke, which severely affected the ADL of stroke patients, and directly affected the rehabilitation outcome of stroke patients. As a treatment method of traditional Chinese medicine, electro-acupuncture has been proven to help improve cognitive dysfunction after stroke. But its mechanism is currently unclear. In recent years, many animal experiments have carried out to explore the mechanism of electro-acupuncture in the treatment of cognitive dysfunction after stroke. However, some research results are contradictory, and some research results are one-sided. Therefore, it is necessary to systematically review the existing results to clarify the effective of electroacupuncture in the treatment of PSCI. The results of this study will provide an evidence for the clinical use of electroacupuncture to treat cognitive dysfunction after stroke.

## Acknowledgments

We would like to acknowledge Jing Li and her team at Chinese Cochrane Centre for their design assistance.

## Author contributions

**Conceptualization:** Tao Wang.

**Funding acquisition:** Guanli Xie, Jianglong Liao, Tao Wang.

**Investigation:** Changfei Yuan, Xiaohan Zhou.

**Methodology:** Yanfei Xu, Deguang Li, Yibo Xia, Miao Tian, Zhifa Yuan.

**Resources:** Wen Luo, Zhe Wang, Changfei Yuan, Yibo Xia, Xiaohan Zhou, Miao Tian, Zhifa Yuan, Tao Wang.

**Writing – original draft:** Guanli Xie, Xiaoxia Tang, Wen Luo, Yanfei Xu, Deguang Li, Zhe Wang, Changfei Yuan, Yibo Xia, Xiaohan Zhou, Miao Tian, Zhifa Yuan, Jianglong Liao, Tao Wang.

**Writing – review & editing:** Guanli Xie, Xiaoxia Tang, Wen Luo, Yanfei Xu, Deguang Li, Zhe Wang, Changfei Yuan, Yibo Xia, Xiaohan Zhou, Miao Tian, Zhifa Yuan, Jianglong Liao, Tao Wang.

## Supplementary Material

Supplemental Digital Content
